# 232. MDL-001: A Broad-Spectrum Antiviral Targeting the Thumb-1 Domain of Viral Polymerases

**DOI:** 10.1093/ofid/ofaf695.084

**Published:** 2026-01-11

**Authors:** Virgil Woods, Tyler Umansky, Sean Russell, David Garvey, David Smith, Daniel Haders

**Affiliations:** Model Medicines, La Jolla, California; Model Medicines, La Jolla, California; Model Medicines, La Jolla, California; DSG Pharma Consulting, LLC, La Jolla, California; University of California, San Diego, San Diego, California; Model Medicines, La Jolla, California

## Abstract

**Background:**

COVID-19 revealed the need for safe, orally available broad-spectrum antivirals that can be deployed when a new pathogen emerges. While RNA-dependent RNA polymerase (RdRp) inhibitors have been effective for some viral families, their broad application has been limited by structural variability and pharmacokinetic challenges. We identified the Thumb-1 domain as a conserved allosteric target in multiple single-stranded RNA (+ssRNA) viruses and developed MDL-001, a potent direct-acting antiviral that exploits this uncharacterized site.

**Figure:**
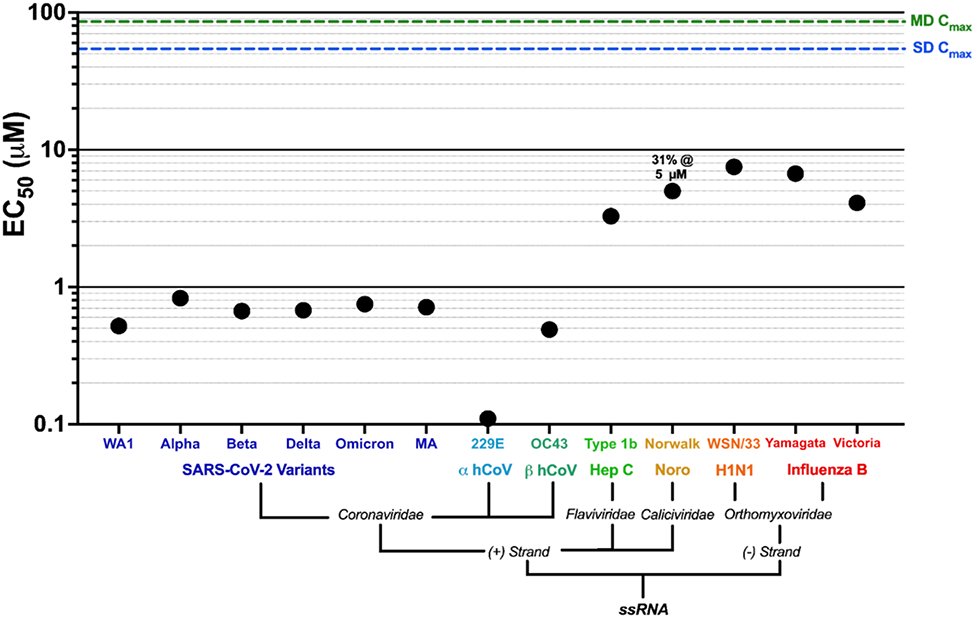
Family tree diagram of 4 viral families, 13 viruses and variants for which MDL-001 exhibited sub 10 µM antiviral activity.

The top EC50 values obtained for each virus/strain/variant obtained across 29 assays are plotted. Complete results including SI50 calculations can be found in Supplementary table S1. MDL-001 Lung, Cmax concentrations in mice after Multiple Dose (MD) and Single Dose (SD) of 250 mg/kg QD dosing are shown as green and blue dashed lines, respectively.

**Methods:**

A comparative structural analysis of RdRp across viral families was performed, integrating homology modeling, multiple sequence alignments, and in silico screening via ChemPrint™, a molecular-geometric deep learning (Mol-GDL) model. MDL-001 was identified as a Thumb-1 inhibitor and assessed for antiviral efficacy in vitro and in vivo. Viral replication assays evaluated its potency against SARS-CoV-2, HCV, Norovirus, and other +ssRNA viruses. Pharmacokinetic (PK) and tolerability studies were conducted in rodents to assess oral bioavailability and systemic distribution.

**Results:**

MDL-001 demonstrated submicromolar potency across multiple viral families, including SARS-CoV-2 variants, with superior selectivity indices relative to standard-of-care antivirals. In a mouse model of SARS-CoV-2 infection, MDL-001 significantly reduced viral lung titers and ameliorated disease progression dose-dependently, achieving efficacy comparable to remdesivir but with an oral administration route. PK studies confirmed favorable lung tissue accumulation, with levels exceeding the in vitro EC90. MDL-001 was well tolerated in preclinical models, with no observed toxicity at therapeutic doses.

**Conclusion:**

These findings establish the Thumb-1 domain as a viable target for broad-spectrum antiviral development. MDL-001 represents a promising, orally available candidate for pandemic preparedness, warranting further clinical investigation.

**Disclosures:**

All Authors: No reported disclosures

